# An Enhanced Informed Watermarking Scheme Using the Posterior Hidden Markov Model

**DOI:** 10.1155/2014/345892

**Published:** 2014-01-16

**Authors:** Chuntao Wang

**Affiliations:** ^1^College of Information, South China Agricultural University, Guangzhou 510642, China; ^2^School of Information Science and Technology, Sun Yat-sen University, Guangzhou 510275, China

## Abstract

Designing a practical watermarking scheme with high robustness, feasible imperceptibility, and large capacity remains one of the most important research topics in robust watermarking. This paper presents a posterior hidden Markov model (HMM-) based informed image watermarking scheme, which well enhances the practicability of the prior-HMM-based informed watermarking with favorable robustness, imperceptibility, and capacity. To make the encoder and decoder use the (nearly) identical posterior HMM, each cover image at the encoder and each received image at the decoder are attacked with JPEG compression at an equivalently small quality factor (QF). The attacked images are then employed to estimate HMM parameter sets for both the encoder and decoder, respectively. Numerical simulations show that a small QF of 5 is an optimum setting for practical use. Based on this posterior HMM, we develop an enhanced posterior-HMM-based informed watermarking scheme. Extensive experimental simulations show that the proposed scheme is comparable to its prior counterpart in which the HMM is estimated with the original image, but it avoids the transmission of the prior HMM from the encoder to the decoder. This thus well enhances the practical application of HMM-based informed watermarking systems. Also, it is demonstrated that the proposed scheme has the robustness comparable to the state-of-the-art with significantly reduced computation time.

## 1. Introduction

Informed watermarking is a kind of technique that adapts watermark signals to host ones, aiming at “eliminating” interferences of host signals on watermarks. This technique originates from the connection between digital watermarking and the problem of communication with side information at the encoder [1–3]. By lying on the consolidated information theories handling communication channels with side information [4, 5] and their adaption to the watermarking case [6–11], this connection makes digital watermarking be promising to achieve a large capacity, high robustness, and good imperceptibility, as demonstrated in the schemes of [6, 12–21].

The scheme of [6] developed by Chen and Wornell is one of pioneering works designing practical informed watermarking algorithms. They constructed a class of lattice-code-based quantization index modulation (QIM) and theoretically proved the optimum of the QIM with distortion compensation (DC-QIM) under the framework of dirty-paper coding [5]. The QIM-based watermarking scheme has the characteristics of high capacity and easy implementation. However, it is weak to scaling attacks that multiply amplitudes of watermarked images with a scalar factor, which is also called gain attacks. To address such issue, several variants of QIM have been proposed in the literature [14–17]. The scheme developed in [14] inserts a pilot signal to facilitate the estimation of the scalar factor, and then it employs the estimated factor to correct amplitudes. The work discussed in [15] resists against gain attacks by designing a gain-invariant step-size for quantization at both the embedder and receiver. Rather than directly quantizing host signals, the approaches proposed in [16, 17] first group a number of host signals into a vector, then take any two vectors to generate an angular value, and finally quantize angular values to insert the message.

Besides the lattice code, the spherical code is also deployed in informed watermarking to implement the dirty-paper coding. The codewords of spherical code have the same energy and thus lie on the surface of a sphere with a radius equivalent to the codewords' norm. This code gives rise to a hyperconic decoding region that centers in the origin. This in turn allows, from a geometrical viewpoint, a signal point multiplied with a constant scalar factor to stay in the same decoding region. Consequently, it leads to a capability to resist against gain attacks. A number of spherical-code-based informed watermarking approaches have been developed in the literature. In [18], Malvar and Florêncio proposed an improved spread-spectrum watermarking scheme (ISS). They used two spread-spectrum vectors of equienergy to represent message bits 0 and 1 and developed an embedding technique to adapt watermark signals to the host one. As a result, the robustness is significantly improved. In [19], Miller et al. designed another informed watermarking scheme using the spherical code. It consists of two stages, namely, informed coding and informed embedding. In informed coding, a message bit is associated with a coset containing a number of spherical codewords, and an optimum codeword is then chosen to represent the message bit. In informed embedding, the selected codeword is tailored, according to the host signal and the constraints of robustness and distortion, and embedded in the host signal. This aims for putting the watermarked signal in the decoding region of the chosen codeword. A similar informed watermarking scheme is presented in [20], in which orthogonal and biorthogonal spherical codewords are used for implementing informed coding and an optimization approach is employed for performing informed embedding. Both schemes of [19, 20] achieve a large embedding capacity as well as high robustness to gain attacks, additive white Gaussian noises (AWGNs), JPEG compression, and so forth. Nevertheless, the scheme of [19] merely evaluated the performance through Monte Carlo simulations, and that of [18] has a relatively high computational complexity because of the employed trellis code with a long codeword length. In [21], Wang et al. developed an informed watermarking using the hidden Markov model (HMM) in the wavelet domain. They used a spherical code with relatively short codeword length for dirty-paper coding, aiming to decrease the computational complexity. This approach achieves comparable performance to the state-of-the-art [19], but it decreases the computational complexity of informed embedding by an order of magnitude.

As the scheme presented in [21] employs the HMM in the wavelet domain to construct the detector, HMM parameters need to be sent as side information to the receiver. This would hinder the practical application of HMM-based informed watermarking systems. To address such issue, we are motivated to develop an informed watermarking scheme using a *posterior* HMM. That is, rather than transmitting HMM parameters to the receiver, we reestimate HMM parameters at the receiver via a particular manner. If the reestimated HMM parameters are sufficiently close to the original one that has been estimated at the transmitter, we thus avoid the transmission of HMM parameters to the receiver. In [22], we had developed a kind of posterior HMM for a spread-spectrum-based robust image watermarking scheme. Considering that the inserted watermark is essentially a weak signal compared to the host one, this scheme takes the watermarked signal as the source to estimate the posterior HMM. Such treatment merely degrades the detection performance slightly, as well demonstrated in [22]. In contrast to the posterior HMM, the one estimated with the original signal is denoted afterwards as the *prior* HMM for notational convenience. 

Although the scheme of [22] shows that it is feasible to estimate the posterior HMM with the watermarked signal, it cannot be directly applied to the case of informed watermarking. As will be illustrated in Section 3, using the posterior HMM for watermark detection would decrease the performance significantly. This is due to the fact that the predefined robustness in informed watermarking is ensured with respect to the prior HMM, but it would be no longer guaranteed if the posterior HMM rather than the prior one is used. The more the differences between the prior and posterior HMMs, the larger the performance degradation is. To make the posterior-HMM-based informed watermarking feasible, the key point is to let both the encoder and decoder adopt HMMs that are sufficiently close to each other. To this end, we impose the same noises that are much stronger than the inserted watermark on the original and received signals and then use the corresponding noisy versions to estimate HMM parameters for the encoder and decoder, respectively. In this way, the estimated HMM at the decoder would be sufficiently close to that at the encoder. Consequently, the decoding performance using this posterior HMM would not degrade or merely degrade slightly.

In the interest of obtaining high robustness to JPEG compression, we take JPEG compression at a small quality factor (QF) as strong noises for the posterior HMM estimation. We then reckon on numerical simulations to determine a practically-optimum QF, namely, QF_opt_. Based on this QF, we construct a posterior-HMM-based informed watermarking scheme. At the encoder, we impose JPEG compression with QF = QF_opt_ on the original image to estimate HMM parameters and use these parameters to implement the informed watermarking algorithm in [21]. At the decoder, we employ the same method as the encoder to obtain HMM parameters and apply them to extract the message. Extensive experimental simulations show that the proposed posterior-HMM-based informed watermarking scheme can achieve the same performance as the prior-based one when attacks are either (equivalently) weaker than or much stronger than the predefined robustness, but it degrades remarkably for other cases. It is also observed that the proposed scheme obtains the robustness comparable to the state-of-the-art [19] with significantly decreased computation time.

The rest of the paper is organized as follows.  Section 2 reviews the HMM in the wavelet domain and the HMM-based informed watermarking scheme presented in [21]. The posterior-HMM-based informed watermarking algorithm is developed in Section 3.  Section 4 introduces the determination of a practically optimum QF by the way of numerical simulations. Based on this QF, experimental simulations are then carried out to evaluate the proposed scheme in Section 5. The conclusion is finally drawn in Section 6.

## 2. Review of the HMM-Based Informed Watermarking Scheme 

As the algorithm proposed in this paper is an enhanced version of that in [21], in this section we briefly review the HMM in the wavelet domain and the HMM-based informed watermarking scheme in [21]. Below are the details.

### 2.1. HMM in the Wavelet Domain

In [23], Crouse et al. proposed an HMM in the wavelet domain (WD-HMM) to characterize the statistical dependency of wavelet coefficients across scales. In [24], Ni et al. used the vector HMM to further capture the cross correlation among subband coefficients in different orientations at the same scale. They denoted it as the vector WD-HMM (VWD-HMM).

**Figure 1:**
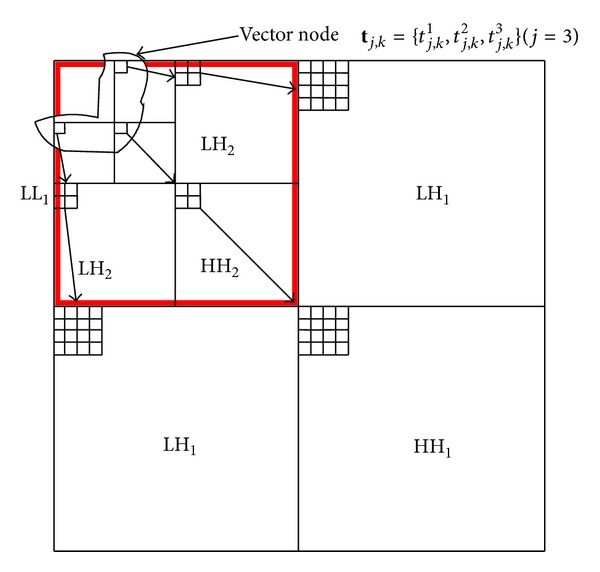
Illustration of *J* = 3 wavelet pyramid decomposition, the VWD-HMM, and a vector quad-tree of wavelet coefficients (3 levels). The square block with thick solid lines denotes the LL_1_ subband at the finest pyramid level, which is further decomposed to form the subbands of LH_2_, HL_2_, HH_2_, and LL_2_ at the second level. And so do the four subbands at the third level.

As supposed in both schemes of [23, 24], any input image is decomposed via orthogonal or biorthogonal wavelets into a *J*-level (*J* ≥ 1) pyramid. The input image is first decomposed into four subbands of LH_1_, HL_1_, HH_1_, and LL_1_. The LL_1_ subband is further used to generate another four subbands of LH_2_, HL_2_, HH_2_, and LL_2_ at the next scale. Such decomposition is repeated until a predefined scale, *J*, is achieved or the subband size does not allow further decomposition, as illustrated in Figure 1.

Figure 1 also illustrates the VWD-HMM. Suppose that *t*_*j*,*k*_^*o*^ stands for the *k*th  (*k* = 1,2,…) wavelet coefficient of the subband at orientation *o*  (*o* = 1, 2, 3) and scale *j*  (1 ≤ *j* ≤ *J*), where *o* = 1, 2, and 3 represent the horizontal, vertical, and diagonal orientations, respectively. Group all *t*_*j*,*k*_^*o*^s with the same *k* and *j* together, resulting in a vector node, **t**_*j*,*k*_ = (*t*_*j*,*k*_^1^ *t*_*j*,*k*_^2^ *t*_*j*,*k*_^3^)^*τ*^, as shown in Figure 1. The superscript “^*τ*^” denotes the matrix transposition. Suppose that **t**_*j*,*k*_ has *M* hidden states *S*_*j*,*k*_ = *m*  (*m* = 1,…, *M*). If each has a state probability *p*_*S*_*j*,*k*__(*S*_*j*,*k*_ = *m*) = *p*_*j*,*k*_^(*m*)^, then ∑_*m*=1_^*M*^ *p*_*j*,*k*_^(*m*)^ = 1. As pointed out in [21, 23, 24], it is reasonable to adopt the same *M* = 2 states, *S*_*j*_ = *m*  (*m* = 1, 2), for all wavelet coefficients at the same scale. One corresponds to small and the other to large wavelet coefficients.

Considering that the average of wavelet coefficients is zero, the probabilistic density function (pdf) of **t**_*j*,*k*_ conditioned on *S*_*j*_ = *m*  (*m* = 1,2) can be modeled as a mixture of two multivariable Gaussians with zero means and covariances of **C**_*j*_^(1)^ and **C**_*j*_^(2)^ [21, 24] as follows:

(1) fj(tj,k)=pj(1)g(tj,k;Cj(1))+pj(2)g(tj,k;Cj(2)),

where *p*_*j*_^(*m*)^ = *p*_*j*,*k*_^(*m*)^ holds for all *k*s, and *g*(**t**; **C**) is defined as

(2) g(t;C)=exp(−(1/2)tτC−1t)(2π)3|det(C)|  ,

where **C** = *E*[**t** **t** ^*τ*^] is a covariance matrix, det(**C**) denotes the determinant of **C**, and |·| stands for the absolute value.

As the parent vector node links itself to its four child vector nodes, as shown in Figure 1, the VWD-HMM uses the Markov chain to capture the energy dependency across scales. To this end, it defines the following state transition probability:

(3) Hj=(pj1→1  pj1→2pj2→1  pj2→2), j=J−1,…,1,

where *p*_*j*_^*m*→*n*^ represents the probability that child vector nodes are in state *n*  (*n* = 1, 2) given that their parent vector node is in state *m*  (*m* = 1,2). Thus, the state probability of child vector nodes can be determined as follows:

(4) pj=pj+1Hj=⋯=pJHJ−1HJ−2⋯Hj,j=J−1,J−2,…,1,

where **p**_*j*_ = (*p*_*j*_^(1)^ *p*_*j*_^(2)^)^*τ*^. Therefore, the VWD-HMM for wavelet coefficients of an image is represented with the following parameter set:

(5) Θ={pJ,HJ−1,…,H1;Cj(m),(j=J,…,1;  m=1,2)}.

According to [24], the Θ can be efficiently estimated by the expectation-maximization (EM) algorithm.

### 2.2. HMM-Based Informed Watermarking Scheme

**Figure 2:**
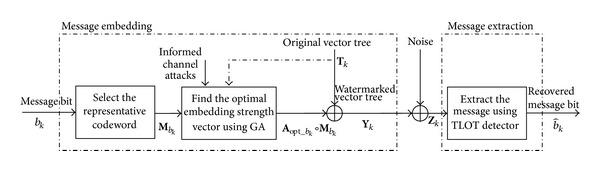
Block diagram of watermark embedding and extraction processes in [21]. The GA, “∘,” and TLOT denote the genetic algorithm, element-wise multiplication, and Taylor-series-approximated locally-optimum test, respectively.

Based on the aforementioned VWD-HMM, the authors in [21] developed an informed watermarking scheme, as illustrated in Figure 2. It includes message embedding and message extraction.

The message embedding is performed as follows.

1. Decompose the original image *I*(*x*, *y*) of size *L*_1_ × *L*_2_ with the biorthogonal 9/7 wavelet into a 3-level wavelet pyramid, and use the coarsest two levels to construct (*L*_1_ *L*_2_/64) vector trees **T**_*k*_ = {**t**_3,*k*_, **t**_2,4*k*−3_, **t**_2,4*k*−2_, **t**_2,4*k*−1_, **t**_2,4*k*_}  (*i* = 1,…, *L*_1_ *L*_2_/64) (see also Figure 1).
2. Given that each vector tree is embedded with one bit, then generate a message sequence **b** of (*L*_1_ *L*_2_/64) random bits. After permuting **b** with a secret key KEY, allocate one permuted bit *b*_*k*_  (*b*_*k*_ = 0,1) to each vector tree. This leads to an information rate of 1/64 bit/pixel.
3. Associate the given message bit *b*_*k*_ to its representative spherical codeword **M**_*b*_*k*__ in coset Coset_*b*_*k*__. The codeword design is recommended to refer to [21].
4. Determine the optimal strength vector **A**_opt_*b*_*k*__ for **M**_*b*_*k*__ through informed embedding. This is formulated as an optimization problem and solved by the genetic algorithm (GA), as given in [21]. In this process, an HMM-based robustness metric is exploited to ensure the predefined robustness, where the HMM is estimated with the original image.
5. Embed (**A**_opt_*b*_*k*__∘**M**_*b*_*k*__) into **T**_*k*_ via the rule **Y**_*k*_ = **T**_*k*_ + **A**_opt_*b*_*k*__∘**M**_*b*_*k*__, where “∘” denotes the element-wise multiplication.
6. After finishing embedding all message bits into their corresponding vector trees via Steps (3) to (5), perform the inverse wavelet transformation to obtain the watermarked image **I** ^*w*^(*x*, *y*).

The message extraction process is implemented in the following manner. For each vector tree **Z**_*k*_  (*i* = 1, 2, …, *L*_1_ *L*_2_/64) at the receiver, the Taylor series-approximated locally-optimum test (TLOT-) based detector is used to find a codeword with the maximum TLOT value, say **M**_*b*_*k*_^*r*^_ ∈ {**M**_0_, **M**_1_}  (*b*_*k*_^*r*^ = 0,1), where the TLOT-based detector exploits the prior HMM. The corresponding coset index (0 or 1) of **M**_*b*_*k*_^*r*^_ is then taken as the extracted message bit *b*_*k*_^*r*^ ∈ {0,1}. After all vector trees have been processed, the extracted bit sequence is reordered with key KEY, and the message sequence **b** ^*r*^ is finally recovered.

Because of the paper length limit, more details of this reviewed HMM-based informed watermarking scheme are recommended to refer to [21]. From the above descriptions, it is clearly found that both the encoder and decoder use the prior VWD-HMM. This implies that the encoder needs to send the prior VWD-HMM to the receiver for message extraction. This would probably hinder the practical application of HMM-based informed watermarking systems. Addressing such problem gives rise to the proposed scheme, as presented in the next section.

## 3. Posterior-HMM-Based Informed Watermarking Scheme

As pointed out in Section 2.2, the prior VWD-HMM parameters need to be sent as side information to the receiver. To handle this problem, we are inspired to take the posterior VWD-HMM estimated with the received image for message extraction, as similarly implemented in [22]. To assess its feasibility, we perform the following examination.

**Figure 3:**
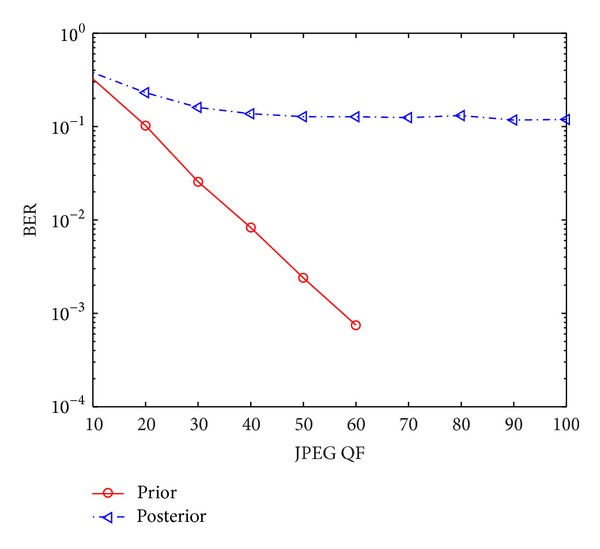
Performance comparison between the prior- and posterior-HMM-based informed watermarking schemes.

In the examination, we test 35 256 × 256 grey images with different textures. For each image, we take JPEG compression with QF = 70 as the predefined robustness and embed the same message sequence of 1024 random bits via the approach in [21] (see also Section 2). The generated watermarked images are then attacked with JPEG compression at different QFs, and the message sequence is extracted from the attacked images. In message extraction, the prior and posterior HMMs that are estimated with the original and attacked images are adopted, respectively. In other words, the compared two cases have the same setting except the used HMM parameters at the receiver. Their corresponding performance in terms of bit error rate (BER) is plotted in Figure 3, where BERs have been averaged over all 35 test images. It is clearly found that the performance of the posterior-HMM-based informed watermarking scheme degrades significantly. This is strongly contrasted with the results for the noninformed scheme of [22], in which the posterior HMM only leads to a slight degradation of detection performance.

The reasons for the significant degradation are explained as follows. Let *I*(*x*, *y*) and *t*_*j*,*k*_  (*j*, *k* ∈ {1,2…}) be the original image and its corresponding wavelet coefficients at scale *j* and location *k*, respectively. Suppose that *I* ^*r*^(*x*, *y*) and *z*_*j*,*k*_ are the received image and its corresponding wavelet coefficients, respectively. Then *z*_*j*,*k*_ would be remarkably different to   *t*_*j*,*k*_ for relatively large embedding strength is generally adopted for informed watermarking with high capacity and robustness. As a result, the posterior HMM estimated with *z*_*j*,*k*_, say Θ_post_, would significantly deviate from the prior one that is estimated with   *t*_*j*,*k*_, namely, Θ_pri_. In return, the detection performance using Θ_post_ decreases greatly, and the predefined robustness ensured with respect to Θ_pri_ is no longer achieved.

According to the above analyses, the performance degradation is mainly due to the deviation of Θ_post_ from Θ_pri_. If they are sufficiently close to each other, the detection performance would then decrease slightly. That is, the key point for the posterior-HMM-based informed watermarking is to let both the encoder and decoder adopt the (nearly) identical HMM parameters. To achieve this objective, we impose the same strong attacks on the original and received images and then use these attacked versions to estimate HMM parameters for the encoder and decoder, respectively. Once the imposed attack is much stronger than the inserted watermark signal, the HMMs estimated at the encoder and decoder would be close enough to each other.

The feasibility of this strategy can be evaluated as follows. Let Δ_*j*,*k*_^attack^ be the distortion due to a particular strong attack. The attacked version of   *t*_*j*,*k*_ is then calculated as *t*_*j*,*k*_^attack^ =   *t*_*j*,*k*_ + Δ_*j*,*k*_^attack^. These *t*_*j*,*k*_^attack^s are used to yield the HMM parameter set, namely, Θ_pri_^attack^. With respect to Θ_pri_^attack^, the predefined robustness is ensured via the informed embedding algorithm in [21] (see also Section 2.2). Similar to the encoder, the received wavelet coefficients *z*_*j*,*k*_s are also performed with the same strong attack to yield the following attacked version:

(6) zj,kattack=zj,k+Δj,kattack=(tj,k+wmj,k+nj,k)+Δj,kattack,

where *wm*_*j*,*k*_ and *n*_*j*,*k*_ denote the inserted watermark and the noise introduced in transmission. These *z*_*j*,*k*_^attack^s are then used to estimate the HMM parameter set, Θ_post_^attack^. Clearly, if Δ_*j*,*k*_^attack^ is sufficiently large compared to the composite signal of *wm*_*j*,*k*_ + *n*_*j*,*k*_, *z*_*j*,*k*_^attack^ would be sufficiently close to *t*_*j*,*k*_^attack^. Consequently, Θ_post_^attack^ would be close enough to  Θ_pri_^attack^. In return, using Θ_post_^attack^ for watermark detection would only degrade the detection performance slightly. In other words, this strategy allows designing a posterior-HMM-based informed watermarking scheme that would achieve a comparable performance to the prior-HMM-based one but eliminate the transmission of HMM parameters to the receiver.

**Figure 4:**
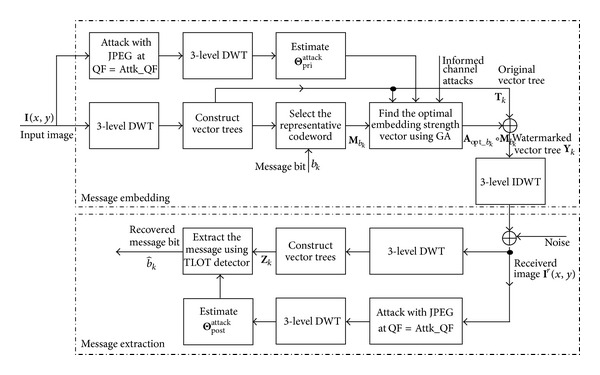
Block diagram of the proposed PostHIW.

In the interest of achieving high robustness against JPEG compression, the prior-HMM-based informed watermarking scheme [21] takes JPEG compression at a particular QF (e.g., QF = 70) as the predefined robustness. Under such setting, we can similarly adopt the JPEG compression with a small QF as the strong attack for estimating the Θ_pri_^attack^ and Θ_post_^attack^. For notational convenience, the QFs for the predefined robustness and the strong attack are denoted as Rbst_QF and Attk_QF, respectively. Therefore, by using the Θ_pri_^attack^ and Θ_post_^attack^ for informed embedding and message extraction, respectively, we can develop a posterior-HMM-based informed watermarking scheme (PostHIW), as illustrated in Figure 4. The details are as follows.

1. Perform Steps (1) to (3) in Section 2.2 to construct vector trees **T**_*k*_(*k* = 1, 2, …, (*L*_1_ *L*_2_)/64), allocate message bits *b*_*k*_, and select representative spherical codeword **M**_*b*_*k*__, respectively.
2. Impose the JPEG compression with QF = Attk_QF on the cover image **I**(*x*, *y*). Then use the EM algorithm (see also Section 2.1) to obtain the HMM parameter set, Θ_pri_^attack^.
3. Set the predefined robustness represented by a JPEG QF to be Rbst_QF.
4. Execute Steps (4) to (6) in Section 2.2 to yield the watermarked image, **I** ^*w*^(*x*, *y*). In this implementation, the Θ_pri_^attack^ estimated with the attacked original image rather than the Θ_pri_ trained with the original image is used for informed embedding.

The posterior-HMM-based extraction process is actually the same as that in Section 2.2 except that the posterior HMM parameter set Θ_post_^attack^ rather than the prior one Θ_pri_ is exploited for watermark detection. In particular, the received (probably polluted) image **I** ^*r*^(*x*, *y*) is firstly attacked with JPEG compression at QF = Attk_QF. The attacked image is then used to estimate the HMM parameter set, Θ_post_^attack^. The Θ_post_^attack^ is finally employed to extract the message, namely, **b** ^*r*^, via the detection approach in Section 2.2.

As aforementioned, the performance of the proposed PostHIW is highly related to the closeness between the Θ_pri_^attack^ and Θ_post_^attack^. The closer the Θ_pri_^attack^ and Θ_post_^attack^ are, the smaller the performance degradation is. Therefore, we need to find an optimum Attk_QF that makes the Θ_post_^attack^ be closest to the Θ_pri_^attack^. This is a fundamental issue for the proposed PostHIW. Its practical determination is given in the next section.

## 4. Determination of the Practically Optimum Attk_QF

As mentioned in Section 3, the setting of Attk_QF is a key parameter for the estimation of Θ_pri_^attack^ and Θ_post_^attack^, and would have a significant impact on the PostHIW's performance. To achieve the best performance, we attempt to determine an optimum setting for Attk_QF. As analyzed in Section 3, the Attk_QF should be sufficiently small to let Θ_post_^attack^ be close enough to Θ_pri_^attack^.

As it is rather tough to obtain an analytic function to characterize the relationship between the Attk_QF and the deviation of Θ_post_^attack^ from Θ_pri_^attack^, we reckon on the numerical simulation to roughly analyze their relationship. This will give rise to a practically optimum setting for Attk_QF, say Attk_QF_opt_.

In the simulation, we test 35 256 × 256 grey images of different textures. We start from estimating HMM parameter sets, Θ_pri_^attack^s, for original test images. For each test image, we impose JPEG compression with Attk_QFs in the range [5 : 5 : 100], where the second 5 denotes a step, and then employ the EM algorithm [21, 23] (see also Section 2.1) to estimate the Θ_pri_^attack^.

We proceed to estimate HMM parameter sets, Θ_post_^attack^s, for watermarked images. As till now we do not determine the Attk_QF_opt_, we cannot employ the Θ_pri_^attack^ with respect to Attk_QF_opt_ to generate, via the approach in Section 3, watermarked images. Instead, we obtain the watermarked images for evaluation using the prior-HMM-based informed watermarking (PriHIW) in [21] (see also Section 2.2). This makes sense as long as the JPEG compression attack is much stronger than the inserted watermark, for example, the situation of a small Attk_QF. In the implementation, we set the Rbst_QF that represents the predefined robustness to be 70, 80, and 90, respectively, and then employ the PriHIW to generate 105 watermarked images. These are further used to obtain 105 HMM parameter sets, Θ_post_^attack^s, via the same EM algorithm as that for Θ_pri_^attack^s.

To evaluate the deviation of Θ_post_^attack^s from Θ_pri_^attack^s, we adopt the Kullback-Leibler divergence (KLD). Assume that total LEV (e.g., LEV = 2) coarsest levels of a *J*-level (*J* ≥ LEV) wavelet pyramid are used for HMM estimation. Further assume that wavelet coefficients for the estimation of Θ_pri_^attack^ and Θ_post_^attack^ are denoted as **t**_*j*,*k*_^pri^s and **t**_*j*,*k*_^post^s, *j* = *J*, …, *J*-LEV + 1, *k* = 1,…, 256 × 256/2^2*j*^, respectively. The KLD at the *j*th level is then calculated as

(7) KLDj=(ppostattack)j×log(ppostattack)j(ppriattack)j,

where (*p*_pri_^attack^)_*j*_ and (*p*_post_^attack^)_*j*_ are the probabilities of wavelet coefficients at the *j*th level that are computed from Θ_pri_^attack^ and Θ_post_^attack^, respectively, which are calculated as follows (see also Section 2.1). The (*p*_pri_^attack^)_*J*_ and (*p*_post_^attack^)_*J*_ at the top parent level (i.e., *j* = *J*) are calculated as

(8) (ppriattack)J=∑k=1  216/22j∑m=12(pJ(m))priattackg(tJ,kpri;(CJ(m))priattack),(ppostattack)J=∑k=1  216/22j∑m=12(pJ(m))postattackg(tJ,kpost;(CJ(m))postattack),

where (·)_pri_^attack^ and (·)_post_^attack^ denote the parameters belonging to sets Θ_pri_^attack^ and Θ_post_^attack^, respectively, and *g*(**t**; **c**) is defined in (2). According to (3), the (*p*_post_^attack^)_*j*_ and (*p*_pri_^attack^)_*j*_ at other levels (i.e., *J*-LEV + 1 ≤ *j* < *J*) are computed as

(9) (ppriattack)j=∑k=1  216/22j∑m=12∑n=12((pj+1(m))priattack(pjn→m)priattack)×g(tJ,kpri;(CJ(m))priattack),(ppostattack)j=∑k=1  216/22j∑m=12∑n=12((pj+1(m))postattack(pjn→m)postattack)×g(tJ,kpost;(CJ(m))postattack).

After obtaining the KLD_*j*_ for each level of the given image, we finally average them to yield the average distance, that is, KLD_avg_ = ∑_*j*=*J*−LEV+1_^*J*^KLD_*j*_/LEV, to reflect the deviation of Θ_post_^attack^ from Θ_pri_^attack^. Further averaging all KLD_avg_s of all test images yields the statistically averaged distance, say $\bar{\text{KL}{\text{D}}_{\text{avg}}}$. In the statistical sense, the $\bar{\text{KL}{\text{D}}_{\text{avg}}}$ characterizes the relationship between Attk_QF and the deviation of Θ_post_^attack^ from Θ_pri_^attack^.

**Figure 5:**
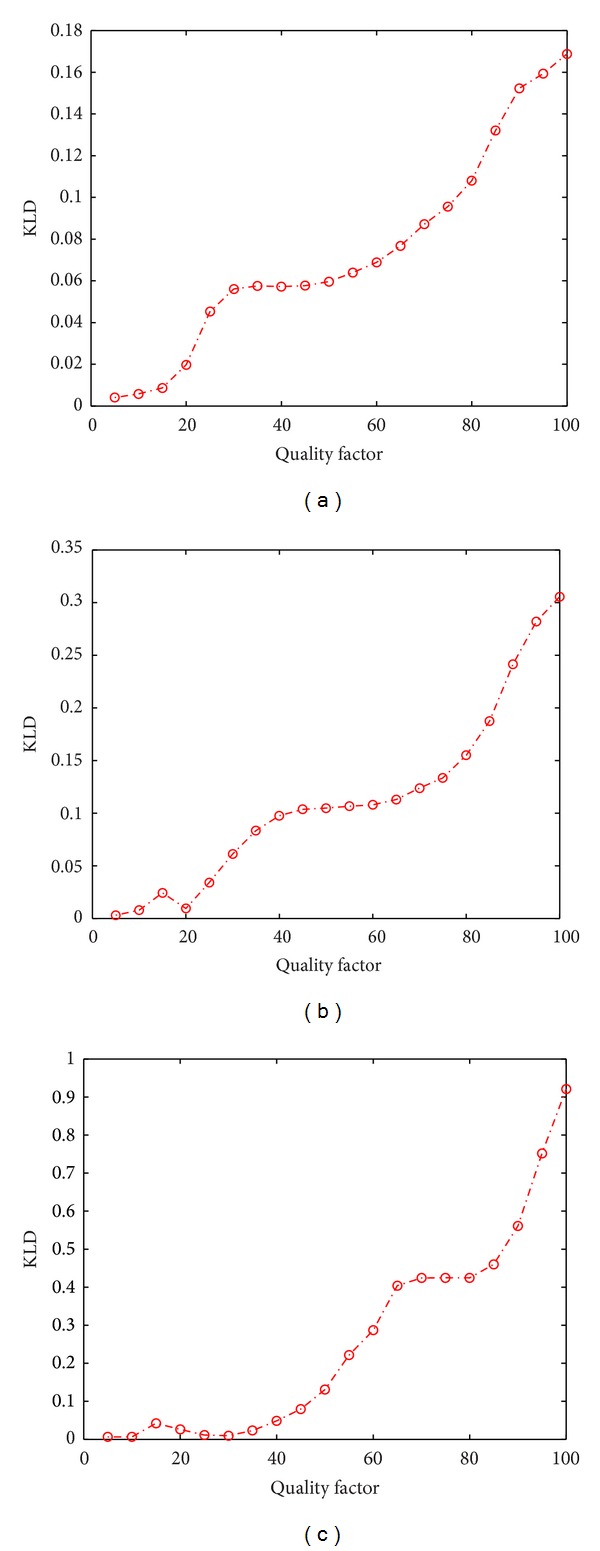
Average KLD between Θ_post_^attack^ and Θ_pri_^attack^ under different predefined robustness settings (a) for Rbst_QF = 70 (b) for Rbst_QF = 80 (c) for Rbst_QF = 90.

Figure 5(a) to Figure 5(c) summarize the relationship between Attk_QF and $\bar{\text{KL}{\text{D}}_{\text{avg}}}$ for Rbst_QF = 70, 80, and 90, respectively. It is observed that increasing the Attk_QF generally increases the $\bar{\text{KL}{\text{D}}_{\text{avg}}}$. This is consistent with the intuition as increasing the Attk_QF would make Δ_*j*,*k*_^attack^ gradually decrease to the same magnitude order of *wm*_*j*,*k*_ + *n*_*j*,*k*_ (see also (6)) and thus Θ_post_^attack^ would gradually deviate from Θ_pri_^attack^. Therefore, it makes sense to take Attk_QF_opt_ = 5 as an optimum setting for the PostHIW using different predefined robustness. Although this setting might not be theoretically ideal, it is really an optimum setting for practical application, as will be well demonstrated in Section 5.

## 5. Experimental Results and Analysis

In this section, we assess the proposed PostHIW by comparing it to the PriHIW [21] (see also Section 2) and the state of the art [19]. Below are the details.

### 5.1. Experimental Setting

In the simulation for the PriHIW and PostHIW, we test 35 256 × 256 grey images with different textures by setting Rbst_QF = 70  and Attk_QF_opt_ = 5, where Rbst_QF and Attk_QF_opt_ represent the predefined robustness and the practically optimum attack parameter for the posterior HMM, respectively. For fair comparison, each test image is embedded, via the PriHIW and PostHIW, with the same message sequence of 1024 random bits, respectively. Their corresponding perceptual distances [21], namely, *D*_DWT_^Pri^ and *D*_DWT_^Post^, respectively, are set to be nearly identical by adjusting the predefined robustness threshold for Rbst_QF = 70.

In the simulation for the state of the art, that is, the trellis-based informed watermarking (TIW) [19], we also embed the same message in each test image. As the TIW presented in [19] is implemented in the DCT domain rather than the wavelet one in our situation, we slightly modify its implementation from the DCT domain to the wavelet one, aiming for fair comparison. That is, we replace embedding units of 12 DCT coefficients and perceptual masks in the TIW with those of 15-node vector trees and visual masks in the PostHIW or PriHIW, respectively. But we keep the other parts of TIW unchanged. Under this setting, the perceptual distance in the wavelet domain can be employed for fair performance evaluation. As set in the comparison between the PriHIW and PostHIW, the perceptual distance for the TIW, namely, *D*_DWT_^TIW^, is also set to be nearly identical to *D*_DWT_^Pri^ and *D*_DWT_^Post^ by adjusting the robustness threshold of TIW. 

### 5.2. Fidelity Evaluation

**Figure 6:**
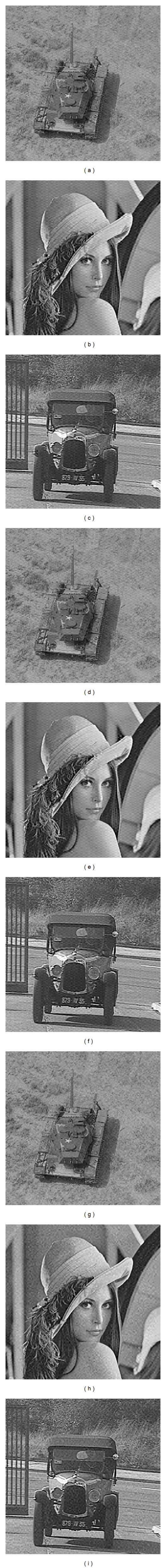
Illustration of the images watermarked by the PriHIW, PostHIW, and TIW. (a) Tank (PSNR = 35.39 dB, *D*_DWT_^Pri^ = 13.81). (b) Lena (PSNR = 33.35 dB, *D*_DWT_^Pri^ = 20.88). (c) Voit (PSNR = 33.11 dB, *D*_DWT_^Pri^ = 21.81). (d) Tank (PSNR = 34.98 dB, *D*_DWT  _^Post^ = 13.81). (e) Lena (PSNR = 33.60 dB, *D*_DWT_^Post^ = 20.90). (f) Voit (PSNR = 33.42 dB, *D*_DWT_^Post^ = 21.83). (g) Tank (PSNR = 35.60 dB, *D*_DWT_^TIW^ = 13.81). (h) Lena (PSNR = 33.81 dB, *D*_DWT_^TIW^ = 20.90). (i) Voit (PSNR = 34.31 dB, *D*_DWT_^TIW^ = 21.81).

Figure 6 illustrates several images watermarked by the PriHIW, PostHIW, and TIW. It can be observed that the subjective visual fidelity of watermarked images of the PriHIW, PostHIW, and TIW is similar to each other.  Table 1 summarizes the mean and standard deviation of peak signal-to-noise ratios (PSNRs) and perceptual distances for all 35 watermarked images generated by the PriHIW, PostHIW, and TIW, respectively. It is seen that these three schemes have nearly identical average perceptual distances.

**Figure 7:**
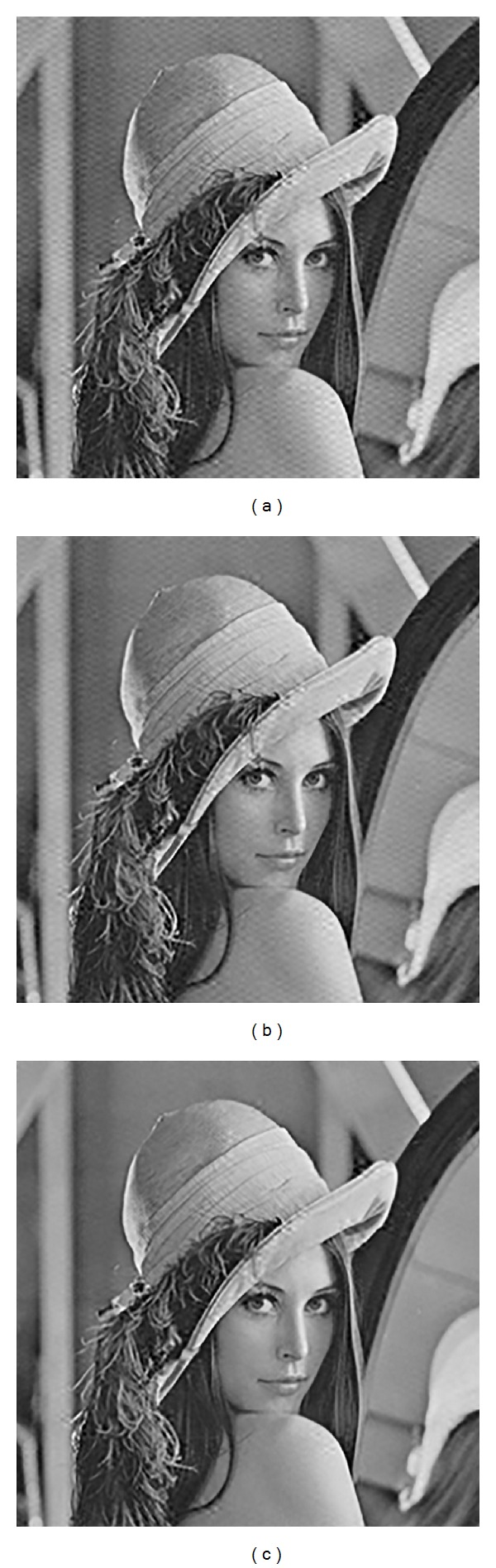
Illustration of the images watermarked by the PostHIW with Rbst_QF set to be 70, 80, and 90, respectively. (a) Lena (Rbst_QF = 70, PSNR = 33.35 dB, *D*_DWT_^Post^ = 20.88). (b) Lena (Rbst_QF = 80, PSNR = 36.09 dB, *D*_DWT_^Post^ = 18.15). (c) Lena (Rbst_QF = 90, PSNR = 39.71 dB, *D*_DWT_^Post^ = 15.32).

In Figure 6, we adopt a relatively large perceptual distance for the convenience of visual artifact illustration. Actually, a smaller perceptual distance and thus the better subjective visual fidelity can be achieved by using a larger Rbst_QF. This is well demonstrated in Figure 7, where the image “Lena” is taken, for example, and the watermarked images are generate, via the PostHIW with the Rbst_QF set to be 70, 80, and 90, respectively.

### 5.3. Performance Comparison between the PriHIW, PostHIW, and TIW

In this subsection, we evaluate the proposed PostHIW by comparing it to the PriHIW and TIW. In the evaluation, we take the watermarked images under the settings of Rbst_QF = 70 and Attk_QF = 5 as test images. On each test image, we impose JPEG compression, gain attacks, additive white Gaussian noise (AWGN), and low-pass Gaussian filtering (LPGF). We then assess the average performance in terms of BER against these attacks. The results are summarized in sections from Section 5.3.1 to Section 5.3.4, followed by the computation time evaluation for all three schemes.

#### 5.3.1. Performance against JPEG Compression

**Figure 8:**
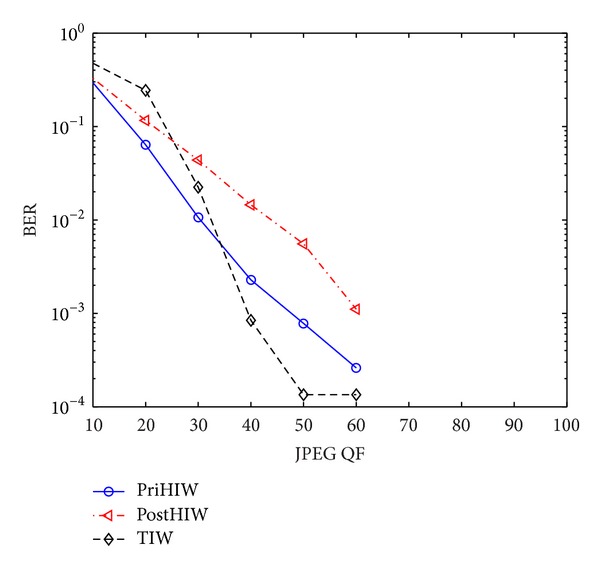
Performance comparison for the PriHIW, PostHIW, and TIW against JPEG compression.

To examine the performance against JPEG compression, we impose JPEG compression with QFs ranging from 10 to 100 on all test watermarked images.  Figure 8 plots the performance comparison for the PriHIW, PostHIW, and TIW. It is shown that the three compared schemes are equivalently robust to JPEG compression with QF ≥ 70. This implies that the predefined robustness indicated by Rbst_QF = 70 has been desirably achieved, which demonstrates the feasibility of the PriHIW and PostHIW.

The PostHIW's performance for QF ∈ (20,70] and QF ≤ 20 is remarkably weaker than and sufficiently close to the PriHIW's one, respectively. These can be analyzed as follows. Suppose that the real HMMs for these attacked images are Θ_real_s. Assume that the KLD between Θ_post_^attack^ (see also Section 4) and Θ_real_ is KLD_post-real_ and the KLD between Θ_pri_ (i.e., estimated with the original image) and Θ_real_ is KLD_pri-real_. When QFs are not sufficiently small, for example, QF ∈ (20,70], both the Θ_pri_ and Θ_post_^attack^ deviate from Θ_real_, but the KLD_pri-real_ is smaller than the KLD_post-real_. Therefore, the PriHIW's detection performance using the Θ_pri_ is better than the PostHIW's one exploiting the Θ_post_^attack^. When QFs are sufficiently small, for example, QF ≤ 20, the KLD_post-real_ and KLD_pri-real_ are probably close to each other, and thus the performance of both the PriHIW and PostHIW is close to each other.

In the comparison with the TIW, the PostHIW has the same robustness as the TIW for QF ≥ 70, achieves worse performance than the TIW for QF ∈ [30,70], and obtains higher robustness for other cases. The reasons are as follows. When QF ≥ 70 holds, the attacks are (equivalently) weaker than the predefined robustness, and thus the performance of BER = 0 can be exactly achieved for all compared schemes. In the situation of QF ∈ [30,70], the attacks are more severe than the predefined robustness of the PostHIW but probably weaker than that of the TIW. As a result, the PostHIW has worse performance than the TIW. This can be expected since the TIW uses the trellis code with long codeword length, which allows achieving better performance at the cost of relatively high computational complexity. In other cases (e.g., QF ≤ 20), the attacks are so strong that both the PostHIW and TIW are subject to uninformed attacks. In contrast to the TIW, the PostHIW well exploits the statistical model of wavelet coefficients and consequently achieves higher robustness than the TIW.

In comparison to the TIW, the PriHIW has the similar performance to the PostHIW except that the PriHIW is worse and better than the TIW for QF ∈ [40,70] and QF ≤ 30, respectively. The reasons are the same as the analysis given above.

#### 5.3.2. Performance against AWGN

**Figure 9:**
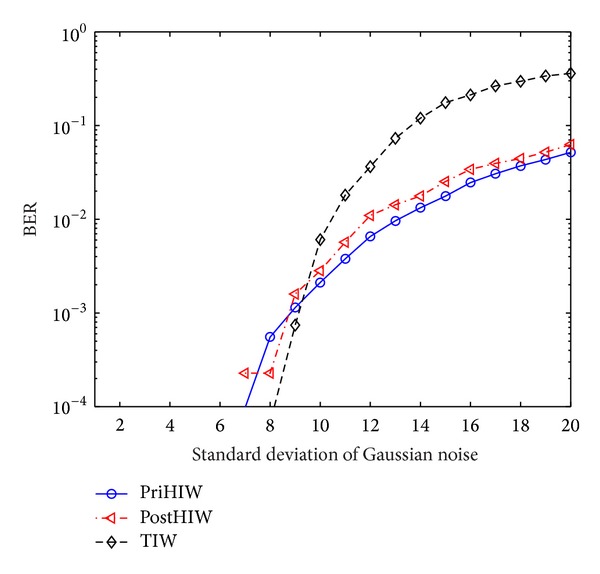
Performance comparison for the PriHIW, PostHIW, and TIW against AWGN.

Figure 9 gives the performance against AWGN attacks for the PriHIW, PostHIW, and TIW, where the standard deviation of Gaussian noise, namely *σ*, is set as the range [1, 20] with step 1. It is seen that both the PriHIW and PostHIW achieve zero BERs for *σ* ≤ 6. This comes from the fact that AWGN attacks with *σ* ≤ 6 are equivalently weaker than the robustness threshold represented by Rbst_QF = 70. The PostHIW obtains a comparable performance to the PriHIW for *σ* ∈ [7,8], where the BERs of the PostHIW for *σ* = 7 and *σ* = 8 are larger and smaller than those of the PriHIW, respectively. This may arise from the unstable HMM estimation for several images. For *σ* ≥ 9, the PostHIW is considerably worse than the PriHIW. The explanations are similar to those for the case QF ∈ (20,70] in the comparison between the PriHIW and PostHIW.

It is observed from Figure 9 that the PostHIW has the same zero BERs as the TIW for *σ* ≤ 6, obtains worse performance than the TIW for *σ* ∈ [7,9], and reaches higher robustness for other cases. The reasons are the same as those in the comparison between the PostHIW and TIW for cases QF ≥ 70, QF ∈ [30,70], and QF ≤ 20, respectively. Similar results are also found for the PriHIW.

#### 5.3.3. Performance against Gain Attacks

**Figure 10:**
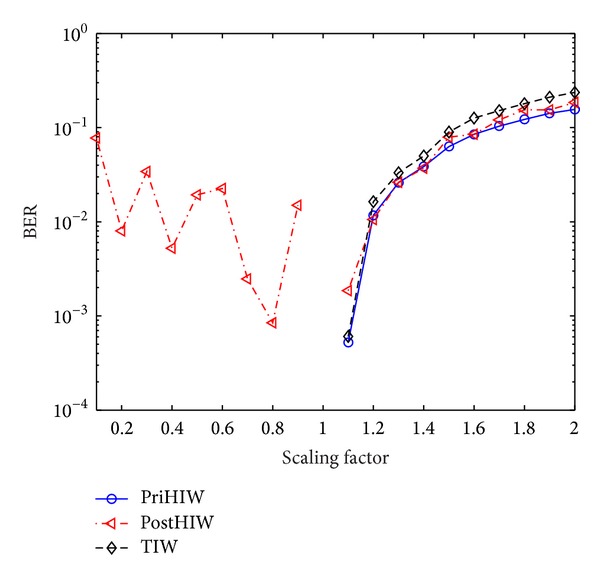
Performance comparison for the PriHIW, PostHIW, and TIW against gain attacks.

As the PriHIW, PostHIW, and TIW employ spherical codes, they are promising to be highly robust to gain attacks. To illustrate this, we impose gain attacks with different scaling factors on the aforementioned test images. In the simulation, scaling factors, say *γ*s, are set as the range [0.1, 2.0] with step 0.1. The performance comparison against these attacks is summarized in Figure 10. It is demonstrated that the performance of the PostHIW is slightly worse than that of the PriHIW for scaling factors varying from 1.1 to 2.0. The reasons are the same as the explanations for the case of QF ≤ 20 in the comparison between the PostHIW and PriHiW. However, the PostHIW is vulnerable to scaling factors below 0.9. This is because these scaling factors significantly decrease the image amplitude so that a large portion of wavelet coefficients are quantized by the JPEG compression attack with Attk_QF = 5 to be zero. In return, this makes, with a large probability, the estimation of Θ_post_^attack^ unstable and consequently degrades the detection performance significantly. In other cases, both the PriHIW and PostHIW have the identical robustness.

It is found from Figure 10 that the PostHIW obtains higher robustness than the TIW for *γ* ≥ 1.2, behaves the same as the TIW for *γ* = 1, and achieves worse performance for other situations. The explanations are similar to those for the cases of QF < 30, QF ≥ 70, and QF ∈ [30,70], respectively, in the comparison between the PostHIW and TIW. Somewhat similar to the PostHIW, the PriHIW is better than and identical to the TIW for *γ* ≥ 1.1 and other cases, respectively.

#### 5.3.4. Performance against LPGF

**Figure 11:**
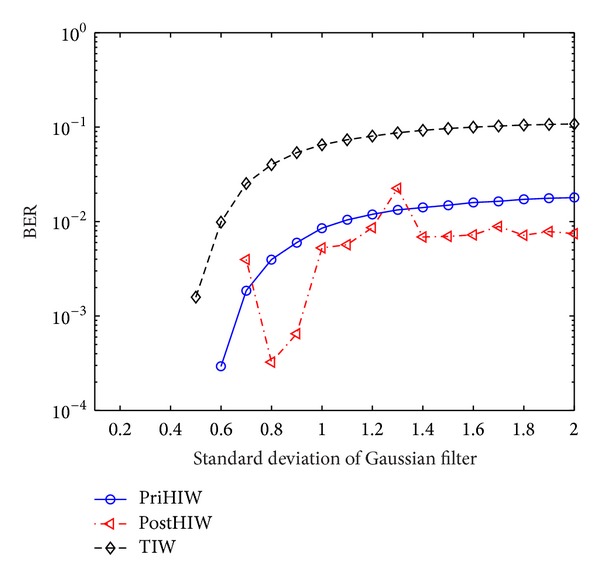
Performance comparison for the PriHIW, PostHIW, and TIW against the LPGF.

We further examine the performance against the LPGF. The standard deviation of Gaussian filter, say *σ*, is set as the range [0.1, 2.0] with step 0.1.  Figure 11 shows the performance comparison for the PriHIW, PostHIW, and TIW.  Figure 11 indicates that the PostHIW is equivalently robust to the PriHIW for *σ* ≤ 0.5, which is due to the fact that these LPGF attacks are actually weaker than or equivalent to the predefined robustness. In other situations, however, the PostHIW is generally better than the PriHIW. This is because LPGF attacks with *σ* ≥ 0.6 would significantly smooth watermarked images, and thus the KLD_post-real_ would be smaller than the KLD_pri-real_ (see also Section 5.3.1). Consequently, the PostHIW using the posterior HMM leads to higher robustness than the PriHIW employing the prior HMM. The BER exceptions for *σ* ∈ {0.7,1.3} may arise from the probably unstable HMM estimation for several watermarked images.

Figure 11 also shows that the PostHIW obtains the same zero BERs as the TIW for *σ* < 0.5, but it yields higher robustness than the TIW for other cases. These can be similarly found for the PriHIW. The analyses are analogous to those in Section 5.3.1.

#### 5.3.5. Computation Time Evaluation

As described in Section 3, the PostHIW replaces the prior HMM with the posterior HMM, but it keeps the other parts of the PriHIW unchanged. Therefore, the computational complexity of both the embedding and detection processes for the PostHIW would be close to that for the PriHIW.

As both the PostHIW and PriHIW use the spherical code with short codeword length, they facilitate the decrease of computational complexity in informed embedding. In contrast, the TIW adopts the trellis-based spherical code with long codeword length, and it also requires Viterbi-decoding-based iterations in the process of informed embedding. Thus, the computational complexity in informed embedding of the TIW would be relatively large. Different from the informed embedding process, however, the detection process of the TIW does not need to perform the time-consuming iterations, in which only one time of Viterbi decoding is needed. Thus, the computational complexity of the detection process would be small.

As it is rather troublesome to obtain an analytic function to characterize the computational complexity of both the embedding and detection processes for the PostHIW/PriHIW and TIW, we rely on the numerical simulation to evaluate their computation time. In the simulation, we implement these schemes with C code and perform them on a 2.2 GHz Intel Core(TM)2 Duo CPU with 2 GB memory. The parameter settings are the same as those in Section 5.1.  Table 2 summarizes the computation time of the embedding and detection processes for the PriHIW, PostHIW, and TIW, respectively, where the results are averaged over all test images. It can be seen that the computation time of the embedding process for both the PriHIW and PostHIW is somewhat close to each other, but it is roughly an order of magnitude lesser than that for the TIW. In addition, it is also observed that the computation time of the detection process for the compared three schemes is approximately in the same order, which can be implemented in real time.

## 6. Conclusion

In this paper, we have presented an enhanced informed image watermarking scheme using the posterior HMM. The key point for this situation is to let both the encoder and decoder obtain (nearly) identical HMM parameter sets. This can be achieved by imposing strong attacks on the original and received images and then using the attacked versions to estimate HMM parameter sets. In the interest of obtaining high robustness to JPEG compression, we take the JPEG compression attack with a small QF as the strong attack. According to numerical simulations, the small QF of 5 can be reasonably considered as an optimum QF for practical use. Based on this setting, we developed a posterior-HMM-based informed watermarking scheme. Extensive simulations show that the proposed posterior-HMM-based informed watermarking scheme is highly robust to the attacks of JPEG compression, AWGN, gain attacks, and LPGF. It is also observed that the proposed scheme is comparable to its prior counterpart but eliminates the transmission of the prior HMM as side information to the receiver. This well enhances the practical application of HMM-based informed watermarking systems. In addition, the proposed scheme is demonstrated to have the performance comparable to the state of the art [19] with significantly reduced computation time.

## Figures and Tables

**Table 1:** Mean and standard deviation (Std.) of watermarked images by different algorithms (Algs.).

Values	Algs.					
	PriHIW		PostHIW		TIW	
	PSNR (dB)	*D*_DWT_^Pri^	PSNR (dB)	*D*_DWT_^Post^	PSNR (dB)	*D*_DWT_^TIW^
Mean	33.46	21.58	33.04	21.59	34.38	21.58
Std.	1.39	8.47	1.40	8.48	1.17	8.48

**Table 2:** Computation time for the embedding and detection processes.

Algorithms	TIW	PriHIW	PostHIW
Embedding (mins)	153.6	3.7	3.8
Detection (secs)	0.18	0.07	0.08
